# KMUP-1 Suppresses RANKL-Induced Osteoclastogenesis and Prevents Ovariectomy-Induced Bone Loss: Roles of MAPKs, Akt, NF-κB and Calcium/Calcineurin/NFATc1 Pathways

**DOI:** 10.1371/journal.pone.0069468

**Published:** 2013-07-25

**Authors:** Shu-Fen Liou, Jong-Hau Hsu, I-Ling Lin, Mei-Ling Ho, Pei-Chuan Hsu, Li-Wen Chen, Ing-Jun Chen, Jwu-Lai Yeh

**Affiliations:** 1 Department of Pharmacy, Chia-Nan University of Pharmacy and Science, Tainan, Taiwan; 2 Department of Paediatrics, Kaohsiung Medical University Hospital, Kaohsiung, Taiwan; 3 Department of Paediatrics, Faculty of Medicine, College of Medicine, Kaohsiung Medical University, Kaohsiung, Taiwan; 4 Department of Medical Laboratory Science and Biotechnology, College of Health Sciences, Kaohsiung Medical University, Kaohsiung, Taiwan; 5 Department and Graduate Institute of Pharmacology, School of Medicine, College of Medicine, Kaohsiung Medical University, Kaohsiung, Taiwan; 6 Departments of Physiology, School of Medicine, College of Medicine, Kaohsiung Medical University, Kaohsiung, Taiwan; University of Pecs Medical School, Hungary

## Abstract

**Background:**

KMUP-1 is a xanthine derivative with inhibitory activities on the phosphodiesterase (PDE) 3,4 and 5 isoenzymes to suppress the degradation of cyclic AMP and cyclic GMP. However, the effects of KMUP-1 on osteoclast differentiation are still unclear. In this study, we investigated whether KMUP-1 inhibits osteoclastogenesis induced by RANKL in RAW 264.7 cells and bone loss induced by ovariectomy in mice, and the underlying mechanisms.

**Principal Findings:**

*In vitro*, KMUP-1 inhibited RANKL-induced TRAP activity, the formation of multinucleated osteoclasts and resorption-pit formation. It also inhibited key mediators of osteoclastogenesis including IL-1β, IL-6, TNF-α and HMGB1. In addition, KMUP-1 inhibited RANKL-induced activation of signaling molecules (Akt, MAPKs, calcium and NF-κB), mRNA expression of osteoclastogensis-associated genes (TRAP, MMP-9, Fra-1, and cathepsin K) and transcription factors (c-Fos and NFATc1). Furthermore, most inhibitory effects of KMUP-1 on RANKL-mediated signal activations were reversed by a protein kinase A inhibitor (H89) and a protein kinase G inhibitor (KT5823). *In vivo*, KMUP-1 prevented loss of bone mineral content, preserved serum alkaline phosphate and reduced serum osteocalcin in ovariectomized mice.

**Conclusions:**

KMUP-1 inhibits RANKL-induced osteoclastogenesis *in vitro* and protects against ovariectomy-induced bone loss *in vivo*. These effects are mediated, at least in part, by cAMP and cGMP pathways. Therefore, KMUP-1 may have a role in pharmacologic therapy of osteoporosis.

## Introduction

The metabolism of bone is dynamic status involving the resorption of bone by osteoclasts and the synthesis of bone matrix by osteoblasts [1]. Factors that increase osteoclast formation or decrease osteoblast formation may enhance the process of osteoporosis. It is known that adenosine 3′,5′-cyclic monophosphate (cAMP) may act as an intracellular second messenger in the process of osteoclastogenesis and osteoblastogenesis [2], [3]. The intracellular level of cAMP is regulated by G protein coupled adenylyl cyclase and its degradation is mediated by the phosphodiesterases (PDEs), a superfamily of enzymes that catalyze the hydrolysis of cAMP and cGMP [4].

The PDE family consists of 11 iso-enzymes, PDE 1 to 11, which differ in their substrate specificity, affinity for cyclic nucleotide, and regulatory properties [5]. Emerging evidence implicates the role of PDE inhibitors in regulation of bone metabolism. For example, cAMP-specific PDE4 inhibitors pentoxifylline and rolipram can increase systemic bone-mass in mice [6], [7]. Other PDE4 inhibitors XT-611 and XT-44 have also been reported to increase osteoblast formation and decrease osteoclast formation in cultured mouse bone-marrow cells, and to prevent bone loss in the animal model of osteopenia [8], [9]. In addition, the cGMP-specific PDE5 inhibitor zaprinast can also inhibit osteoclast formation [10]. Therefore, the PDE inhibitor may be an agent worthy of further investigation in treatment or prevention of osteoporosis.

Receptor activator of NF-κB ligand (RANKL), a tumor necrosis factor (TNF) family cytokine, is essential for osteoclast differentiation. The binding of RANKL to its receptor, RANK, triggers the activation of cytoplasmic tumor necrosis factor receptor-associated factor 6 (TRAF6) [11], which subsequently induces the activation of the NF-κB pathway, Src/phosphatidylinositide (PI) 3-kinase/Akt axis, mitogen activated protein kinases (MAPKs), and transcription factors including AP-1 and NFATc1. These transcription factors are critical for the regulation of genes involved in osteoclast differentiation and bone resorptive activity of mature osteoclasts [12]. Activation of these pathways results in complex responses ultimately causing fusion of osteoclast progenitors into mature multinucleated osteoclasts. These mature osteoclasts express a specific cohort of gene products, including calcitonin receptor, tartrate-resistant acid phosphatase (TRAP), cathepsin K, and matrix metalloproteinase-9 (MMP-9) [13]. Thus, agents suppressing RANKL signaling may inhibit osteoclastogenesis and bone loss.

KMUP-1 (7-[2-[4-(2-chlorophenyl)piperazinyl]ethyl]-1,3-dimethylxanthine), a chemical synthetic xanthine-based derivative, has been demonstrated to inhibit the enzyme activity of PDE3, 4 and 5, suggesting its ability to inhibit metabolism of cAMP and cGMP, resulting in relaxation of smooth muscle in aorta [14], corporeal cavernosa [15] and trachea [16]. In addition, KMUP-1 has been shown to possess multifunctional properties, including anti-inflammatory [17], anti-proliferation [18], [19], cardioprotective [20] and neuroprotective properties [21]. However, the effects of KMUP-1 on osteoclast differentiation and bone resorption have not been reported. In this study, we demonstrated that KMUP-1 had anti-osteoclastogenic and anti-resorptive activities *in vitro* and *in vivo*, and investigated mechanisms underlying these effects.

## Materials and Methods

### Drugs

KMUP-1 hydrochloride (KMUP-1 HCl) was synthesized in our laboratory. MTT (3-[4,5-dimethylthiazol-2-yl]-2,5-diphenyl tetrazolium bromide), H89, KT5823, BAPTA and ionomycin were purchased from Sigma-Aldrich (St. Louis. Mo, USA). Antibodies against IκBα, phospho-IκBα, phospho-ERK, Akt, phospho-Akt, c-Fos and MMP-9 were purchased from Cell Signaling Technology (Beverly MA, USA). Polyclonal antibodies against ERK and phospho-JNK were purchased from Upstate Biotechnology (Lake Placid, NY, USA). Anti-p65 and anti-PARP were purchased from Millipore (Temecula, CA, USA). Monoclonal antibodies against JNK and RANKL were purchased from R&D Systems (Minneapolis, MN, USA). Monoclonal antibody against phospho-p38 was purchased from Abcam (Massachusetts, USA). Anti-calcineurin was purchased from BD Biosciences (San Diego, California, USA). Anti-MMP-2 was purchased from Thermo Scientific (Cheshire, UK). Anti-NFATc1, anti-p38, and anti-HMGB1 were purchased from Santa Cruz Biotechnology (Santa Cruz, CA, USA). Monoclonal antibodies against β-actin and HRP-conjugated secondary antibodies were from Sigma-Aldrich. The TRAP staining kit was obtained from Sigma-Aldrich. Other reagents were purchased from Sigma-Aldrich. KMUP-1 HCl was dissolved in distilled water and sildenafil was dissolved in vehicle (distilled water containing 0.5% methyl cellulose) for experiments.

### Osteoclast Differentiation

The RAW264.7 mouse monocyte/macrophage cell line was purchased from the Bioresource Collection and Research Center in Taiwan. Cells were maintained in DMEM medium supplemented with 10% FBS, 2 mM glutamine, 100 U/ml penicillin G, 100 µg/ml streptomycin and 0.25 mg/ml amphotericin B at 37°C and 5% CO_2_. For differentiation of osteoclasts, RAW264.7 cells (2×10^4^ cells/well, in 24-well plates) were cultured in the presence of RANKL (10 ng/ml) for 5 days. The culture medium was replaced every 3 days.

### Cytotoxicity Assay for KMUP-1

RAW264.7 cells were cultured in 96-well plates and treated with KMUP-1 at various concentrations for 24 or 48 h. MTT solution (0.5 mg/ml) was added and incubated further for 4 h. Then the culture medium was removed, and cells were dissolved in isopropanol and shaken for 10 min. The amount of MTT formazan was quantified by determining the absorbance at 540 nm and 630 nm, using an ELISA reader (DYNEX Technologies, Germany).

### TRAP Activity Assay and TRAP Staining

Osteoclasts were identified by the assay of tartrate-resistant acid phosphatase (TRAP) activity. In brief, TRAP-positive multinucleated cells with more than three nuclei were counted as osteoclasts. RAW264.7 cells were cultured with RANKL (10 ng/ml) in the presence of KMUP-1 for 5 days. After 5 days in culture, cells were washed with PBS and fixed with 10% formaldehyde for 10 min. Fixed cells were subjected to an assay for TRAP activity using 10 mM *p*-nitrophenylphosphate in 50 mM citrate buffer (pH 4.6) and 10 mM of sodium tartrate as a substrate for 30 min. The reaction was stopped with 0.1 N NaOH. The absorbance at 405 nm was determined by a microplate reader. For TRAP staining, cells were fixed with 10% formaldehyde for 10 min, and then stained with 0.01% naphthol AS-MX-phosphate disodium and 0.05% fast red violet LB salt in presence of 50 mM sodium tartrate and 90 mM sodium acetate (pH 5.0). After staining, cells were washed with PBS and TRAP-positive multinucleated osteoclasts (>3 nuclei) were counted under a light microscope.

### Bone Resorption Assay

RAW264.7 cells (10^3^ cells/well) were seeded onto calcium phosphate-coated plates (BD Biosciences, MA) and treated with 10 ng/ml RANKL for 5 days until mature multinucleated osteoclasts were formed. Mature osteoclasts were treated with or without KMUP-1 in the presence of RANKL for 48 h. After the incubation, the cells were removed from the plates with 5% sodium hypochlorite, and pit formation was observed under an optic microscope (Olympus, Tokyo, Japan). The resorbed area was also measured by image analyzer and expressed as percentage (%) of the control value.

### Cytokine Detection by ELISA

RAW264.7 cells (10^5/^ml) were seeded in a 24 well plate. The cells were treated with or without KMUP-1 in the presence of RANKL (10 ng/ml) for 24 h. Then the cell suspensions were collected, and the levels of TNF-α, IL-1β, IL-6 and IL-10 in the cell culture medium were measured using enzyme-linked immunosorbent assay (ELISA) kit according to the manufacturer’s protocol (Pierce Biotechnology, Rockford, IL, USA).

### Real-Time Quantitative Reverse Transcription-PCR

Total RNA was extracted from RAW264.7 cells using SV Total RNA Isolation System by a standard protocol (Promega, Madison, WI, USA). First-strand cDNA was synthesized by Reverse Transcription System Kit (Promega, Madison, WI, USA) according to the manufacturer’s instruction, with 500 ng of total RNA. Real time q-PCR was performed on the ABI Step one Plus System (Applied Biosystems, Foster City, CA, USA) using Power SYBR Green PCR Master Mix (Applied Biosystems). The following primer sets were used: *TRAF6*, 5′-GATCGGGTTGTGTGTGTCTG-3′ (forward) and 5′-AGACACCCCAGCAGCTAAGA-3′ (reverse); *TRAP*, 5′-CTACCTGTGTGGACATGACCA-3′ (forward) and 5′-GCACATAGCCCACACCGTTC-3′ (reverse); *c-Fos*, 5′-ACTTCTTGTTTCCGGC-3′ (forward) and 5′-AGCTTCAGGGTAGGTG-3′ (reverse); *NFATc1*, 5′-CCGTGCTTCCAGAAAATAACA-3′ (forward) and 5′-TGTGGGATGTGAACTCGGAA-3′ (reverse); *MMP-9*, 5′-CTGGACAGCCAGACACTAAAG-3′ (forward) and 5′-CTCGCGGCAAGTCTTCAGAG-3′ (reverse); *Fra-1* 5′-CAGCCTCATTTCCTGGGACC-3′ (forward) and 5′-CCTTTCTTCGGTTTCTGCACT-3′ (reverse); *Fra-2*, 5′-ATCCACGCTCACATCCCTAC-3′ (forward) and 5′-GTTTCTCTCCCTCCGGATTC-3′ (reverse); *c-src*, 5′-CCAGGCTGAGGAGTGGTACT-3′ (forward) and 5′-CAGCTTGCGGATCTTGTAGT-3′ (reverse); *Cathepsin K*, 5′-GAAGAAGACTCACCAGAAGCAG-3′ (forward) and 5′-TCCAGGTTATGGGCAGAGATT-3′ (reverse) and *GAPDH*, 5′-AAATGGTGAAGGTCGGTGTG-3′ (forward) and 5′-TGAAGGGGTCGTTGATGG-3′ (reverse). All reactions were run in triplicate and were normalized to the housekeeping gene glyceraldehyde-3-phosphate dehydrogenase (GAPDH). Relative differences in real-time PCR results were evaluated using the comparative cycle threshold method.

### Gelatin Zymography

MMP-2 and MMP-9 were evaluated by gelatin zymography. The conditioned medium was separated under nonreducing conditions using 10% SDS-PAGE containing 0.1% gelatin as we previously described [22]. After electrophoresis, gels were washed twice for 30 minutes in 1× Zymogram Renaturing Buffer (2.5% [w/v] Triton X-100) to remove SDS and allow the renaturation of MMPs and then incubated overnight in 1× Zymogram Developing Buffer (50 mM Tris-HCl, pH 7.5, 5 mM CaCl_2_, and 0.02% [w/v] Triton X-100). The bands were visualized by staining for 30–60 min with a solution containing 0.1% Coomassie R-250 in 40% ethanol and 10% acetic acid; followed by destaining for 2 h at room temperature in a solution containing 10% ethanol and 7.5% acetic acid. The images were taken using the UVP Biochemi EC3 imaging system (UVP, LLC, Upland, CA).

### Preparation of Cytosolic and Nuclear Protein Extracts

Separation and preparation of cytoplasmic and nuclear extracts were performed using NE-PER Nuclear and Cytoplasmic Extraction kit (Pierce Biotechnology, Rockford, IL) according to the manufacturer’s instructions. All of the fractionated protein solutions were stored at -80°C until analysis.

### Analysis of Calcium Oscillations

RAW264.7 cells were incubated with RANKL (10 ng/ml) for 5 day in the presence or absence of 10 µM KMUP-1. After washing with PBS, cells were incubated with 1 µM fluo-4 AM for 30 min at 37°C and then cells were excited at 488 nm and the fluorescence images with emission at 505–530 nm. For ratiometric measurement of Ca^2+^ in a single cell, the fluorescence intensity of fluo-4 was calculated and expressed as the percent maximum ratio increase, which was obtained by the addition of 1 µM ionomycin at the end of experiments as previously described [23], [24]. BAPTA-AM, a membrane permeable Ca^2+^ chelator, was used to reduce intracellular calcium.

### Western Blot Analysis

Cells were treated with indicated concentrations of KMUP-1 for the indicated times. Reactions were terminated by washing twice with cold PBS. The cells were then harvested and Western analyses were performed as we previously described [20].

### Immunocytochemistry

RAW264.7 cells were pretreated with KMUP-1 (10 µM) for 3 h and then co-treated with RANKL (10 ng/ml) for 30 min or 18 h for the detection of translocation of NF-κB and HMGB1, respectively. Fixation was performed in 10% formaldehyde for 30 min at 4°C. Preparations were rinsed and incubated with mouse anti-NF-κB or rabbit anti-HMGB1 antibody overnight at 4°C. This was followed by incubation with FITC-conjugated secondary antibody and images were collected by confocal laser-scanning microscope (Olympus Fluoview FV1000, Olympus Optical Co, Tokyo, Japan).

### Mice

BALB/c mice (6 to 8-week-old females) from the National Laboratory Animal Breeding and Research Center (Taipei, Taiwan) were housed under conditions of constant temperature and controlled illumination (light on between 7∶30 and 19∶30 hours). Food and water were available ad libitum. The study was approved by the Animal Care and Use Committee of Kaohsiung Medical University.

### Ovariectomy-Induced Osteoporosis

Forty female BALB/c mice (25–30 g) were used for this study. Mice were ovariectomized (OVX) bilaterally under pentobarbitone anaesthesia and control mice were sham-operated (Sham) for comparison. Two days after the operation, the mice were treated with KMUP-1 (1, 5 and 10 mg/kg) orally every day. After 30 days of KMUP-1 treatment, the bone morphometric parameters and micro-architectural properties of the proximal tibias were determined using a microcomputed tomography (µCT) (Skyscan 1076, Belgium). Bone mineral content (BMC), trabecular number (Tb.N.), trabecular separation (Tb.Sp.), trabecular thickness (Tb.Th.) and bone volume/tissue volume (BV/TV) were quantitatively analyzed using a specific software compatible with the µCT system. Furthermore, the blood sample was collected for serum isolation. Serum was separated by centrifugation (1500× g) and then stored at -80°C for analysis of osteocalcin and alkaline phosphatase (ALP).

### Statistical Analysis

Results were expressed as mean ± S.E.M. from at least three independent experiments. Student’s t-test was used for determining the significance of differences between two groups, whereas one-way ANOVA was used for multiple comparisons. P values of less than 0.05 were considered to be statistically significant in all cases.

## Results

### Effects of KMUP-1 on Cell Viability and Proliferation

**Figure 1:**
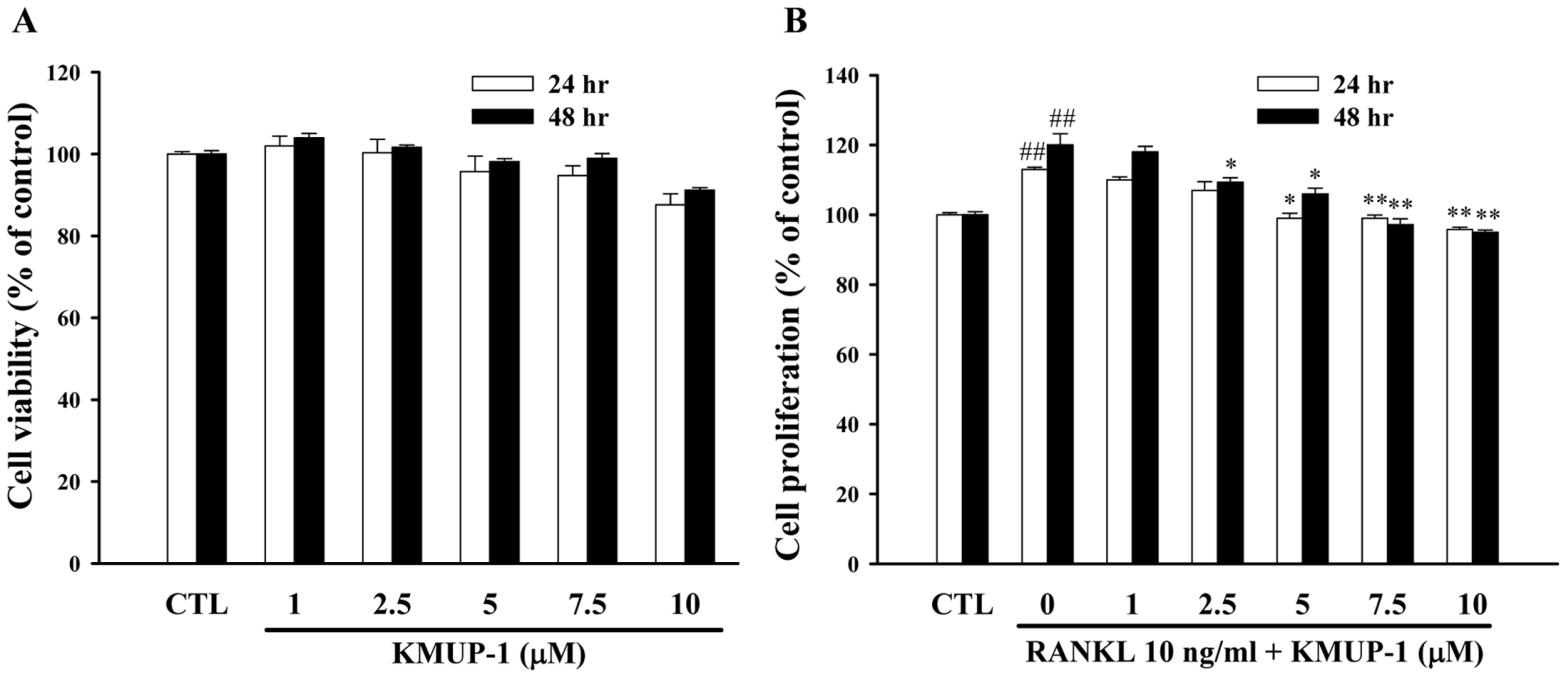
Effects of KMUP-1 on cell viability and proliferation of RAW264.7 cells. (**A**, **B**) Cells were cultured with or without RANKL (10 ng/ml) with various concentrations of KMUP-1 (1–10 µM) for 24 or 48 h. Cell viability and proliferation were determined by the MTT method. Each value represents the mean ± S.E.M. of three independent experiments, with triplicate determinations in each experiment. ^##^ *P*<0.01 compared with control; *P<0.05, **P<0.01 compared with RANKL alone.

Figure 1A showed that KMUP-1 had no significant effect on viability of RAW264.7 cells. The IC50 values of KMUP-1 for 24 h and 48 h were 54.59 µM and 34.40 µM, respectively (data not shown), so we used concentrations below this range (1 to 10 µM) to investigate its effects on proliferation by the MTT assay. We found that RANKL induced cell proliferation, and KMUP-1 inhibited this effect in a concentration-dependent manner (Figure 1B). In addition, we also used the concentrations within this range to investigate the activity against osteoclast differentiation.

### Effects of KMUP-1 on Osteoclastogenesis

**Figure 2:**
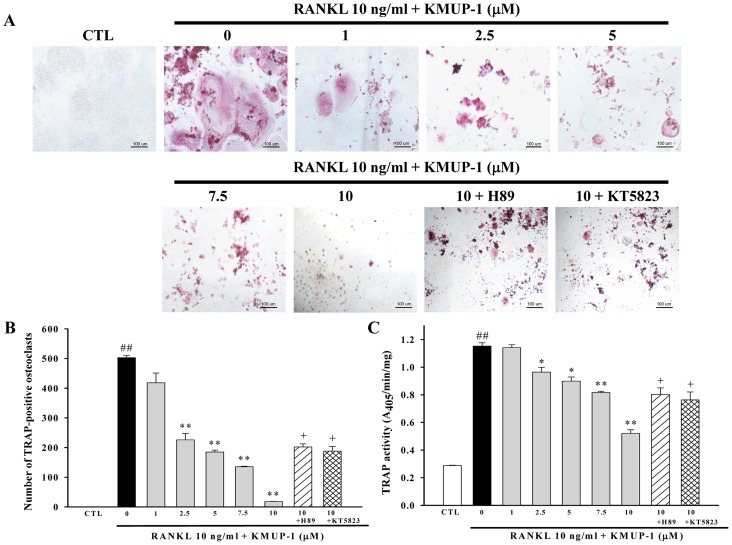
Effects of KMUP-1 on RANKL-induced osteoclastogenesis. RAW264.7 cells were cultured with RANKL (10 ng/ml) for 5 days to induce osteoclast differentiation, with treatment of KMUP-1 (0–10 µM) to assess its anti-osteoclastogenic effects. In addition, cells treated with KMUP-1 (10 µM) had pretreatment of PKA inhibitor H89 (10 µM) and PKG inhibitor KT5823 (3 µM) to determine underlying mechanisms. (**A**) After 5 days of culture, TRAP positive cells and multinucleated osteoclasts were counted under the microscope. (**B**) The number of TRAP-positive multinucleated cells was counted. (**C**) TRAP activity was measured at 405 nm. Each value represents the mean ± S.E.M. of three independent experiments, with triplicate determinations in each experiment. ^##^ *P*<0.01 compared with control; *P<0.05, **P<0.01 compared with RANKL alone; ^+^ *P*<0.05 compared with KMUP-1 10 µM plus RANKL.

RAW264.7 cells were cultured with RANKL (10 ng/ml) to induce osteoclastogenesis. After 5 days of culture, TRAP positive multinucleated osteoclasts were counted under the microscope. We found that KMUP-1 concentration-dependently inhibited differentiation of osteoclasts as evidenced by attenuating TRAP formation in RAW264.7 cells (Figure 2A). Indeed, KMUP-1 inhibited osteoclast differentiation in a concentration-dependent manner with an IC50 of 3.76 µM (Figure 2B). Similarly, KMUP-1 (2.5–10 µM) significantly suppressed TRAP activity (Figure 2C). Notably, all these anti-osteoclastogenic effects were attenuated by a protein kinase A (PKA) inhibitor (H89) and a protein kinase G (PKG) inhibitor (KT5823).

### Effects of KMUP-1 on Bone Resorption

**Figure 3:**
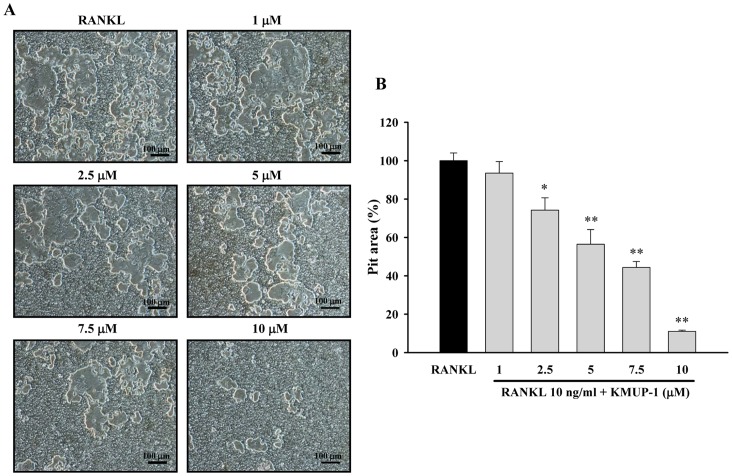
Effects of KMUP-1 on RANKL-induced pit formation in mature osteocalsts. (**A**) Mature osteoclasts were treated with RANKL (10 ng/ml) and KMUP-1 **(**1–10****µM) for 48 h. Pit formation on the disc was observed by optical microscopy. (**B**) Pit areas were quantified using Image Pro Plus analyzer Version 4.6 (Media Cybernetics Inc., MD). Each value represents the mean ± S.E.M. of three independent experiments, with triplicate determinations in each experiment. *P<0.05, **P<0.01 compared with RANKL alone.

We further investigated the effects of KMUP-1 on the bone-resorption activity of mature osteoclasts and found that it also inhibited RANKL-mediated bone resorption, as measured by an *in vitro* model system (Figure 3A). Notably, there was a concentration-dependent inhibition of bone resorption when mature osteoclasts were incubated with KMUP-1 and RANKL for 48 h (Figure 3B).

### Effects of KMUP-1 on Cytokine Production

**Figure 4:**
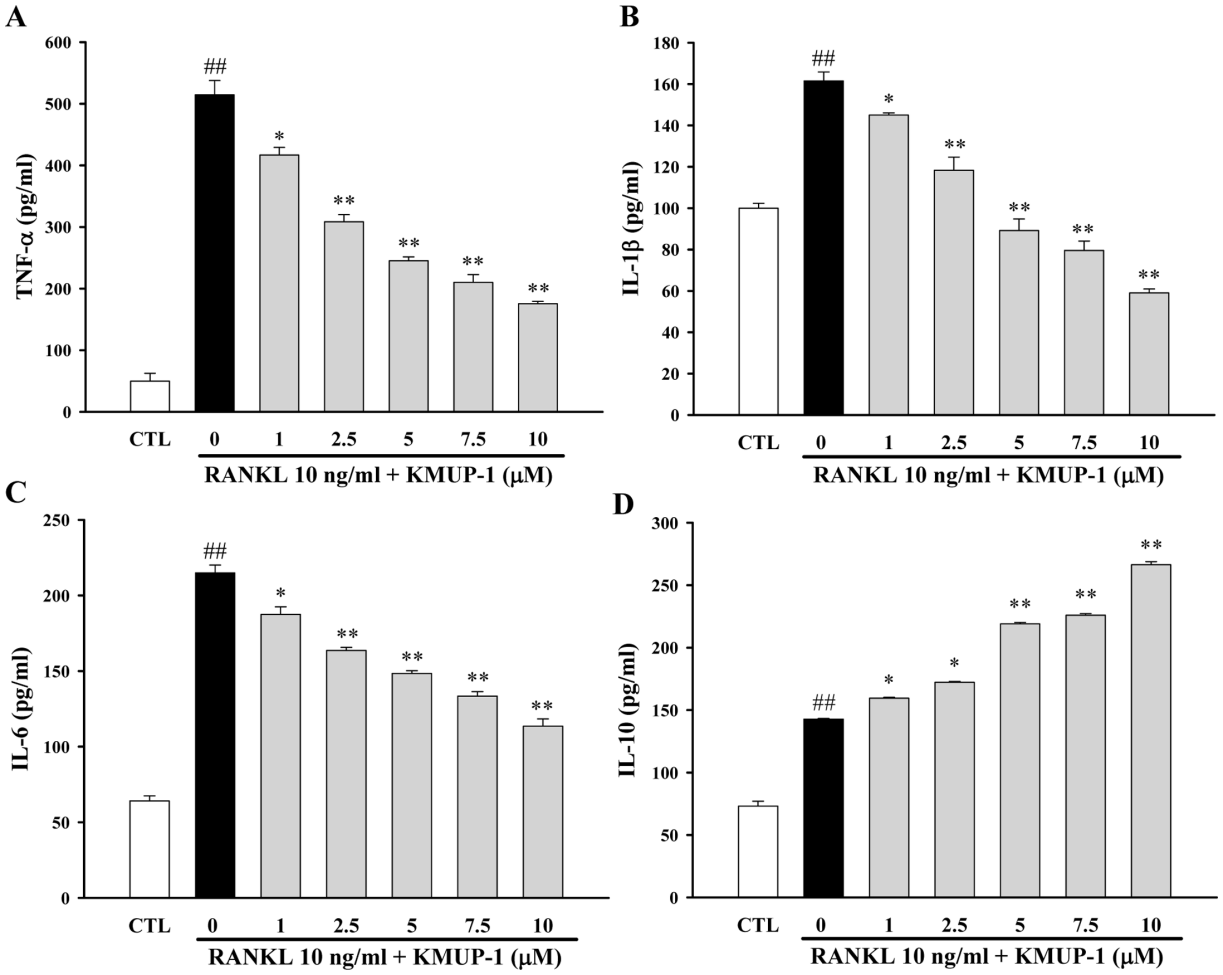
Effects of KMUP-1 on RANKL-induced production of inflammatory cytokines. (**A**) TNF- α, (**B**) IL-1 β, (**C**) IL-6, and (**D**) IL-10 were determined 24 hr after co-incubation with RANKL (10 ng/ml) and KMUP-1 (0–10 µM). Each value represents the mean ± S.E.M. of three independent experiments, with triplicate determinations in each experiment. ^##^ *p*<0.01 compared with control; *P<0.05, **P<0.01 compared with that treated with RANKL alone.

There is growing evidence that inflammation may be one of the causal factors of osteoporosis. Several cytokines such as TNF-α, IL-1β, and IL-6 were implicated in the pathogenesis of osteoporosis. These cytokines are important determinants of osteoclast differentiation and its bone resorptive activity. As shown in Figure 4, all cytokines were relatively low in resting RAW264.7 cells, but markedly increased upon exposure to RANKL. Treatment with KMUP-1 inhibited RANKL-induced TNF-α, IL-1β, and IL-6 production in a concentration-dependent manner (Figure 4A–C). Furthermore, KMUP-1 significantly up-regulated IL-10 production in a concentration-dependent manner (Figure 4D).

### Effects of KMUP-1 on Regulation of Osteoclastogenesis-Associated Genes Are Mediated by cAMP and cGMP

**Figure 5:**
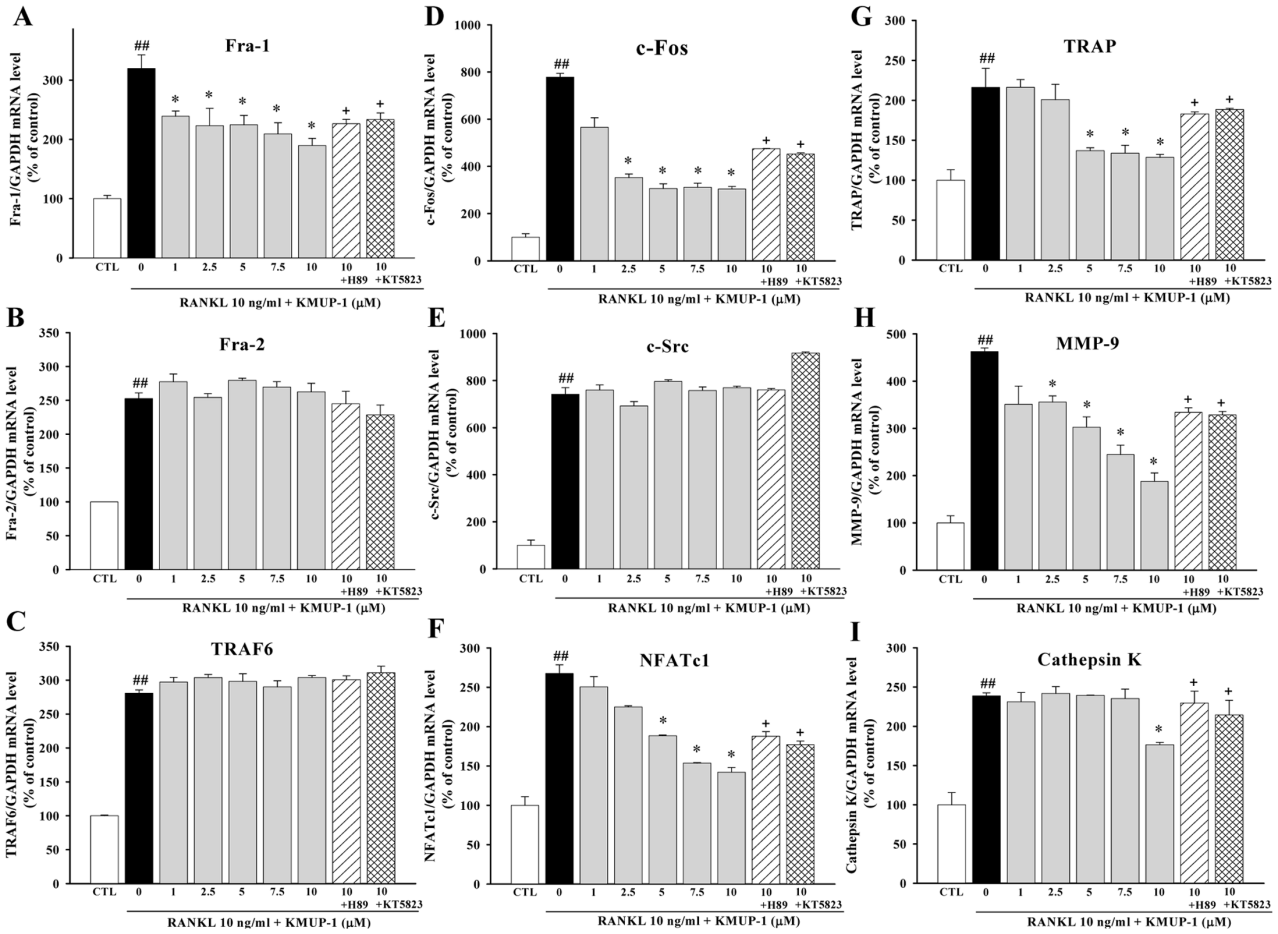
Effects of KMUP-1 on mRNA expression of osteoclastogenesis-related genes. Total RNA was extracted from RAW264.7 cells cultured for 24 h in the presence of RANKL (10 ng/ml) and KMUP-1 (0–10 µM), or with pretreatment of PKA inhibitor H89 (10 µM) and PKG inhibitor KT5823 (3 µM) for 30 min. The mRNA expression of the indicated genes was analyzed by real time RT-PCR. Each value represents the mean ± S.E.M. of three independent experiments, with triplicate determinations in each experiment. ^##^ *P*<0.01 compared with control; *P<0.05 compared with RANKL alone; ^+^ *P*<0.05 compared with KMUP-1 10 µM plus RANKL.

Using real time RT-PCR, we examined the effects of KMUP-1 on gene expression associated with osteoclastogenesis. Figure 5 showed that KMUP-1 decreased mRNA levels of fos-related antigen 1 (Fra-1), c-Fos, NFATc1, TRAP, MMP-9, and Cathepsin K in the presence of RANKL, with no effects on TRAF6, Fra-2 and c-Src. To further determine pathways underlying inhibitory effects of KMUP-1 on these RANKL-mediated signal activations, we conducted experiments using the PKA inhibitor (H89) and the PKG inhibitor (KT5823). As shown in Figure 5, most inhibitory effects of KMUP-1 on RANKL-mediated signal activations were reversed by H89 or KT5823.

### Effects of KMUP-1 on RANKL-induced Activations of MMP-2 and MMP-9

**Figure 6:**
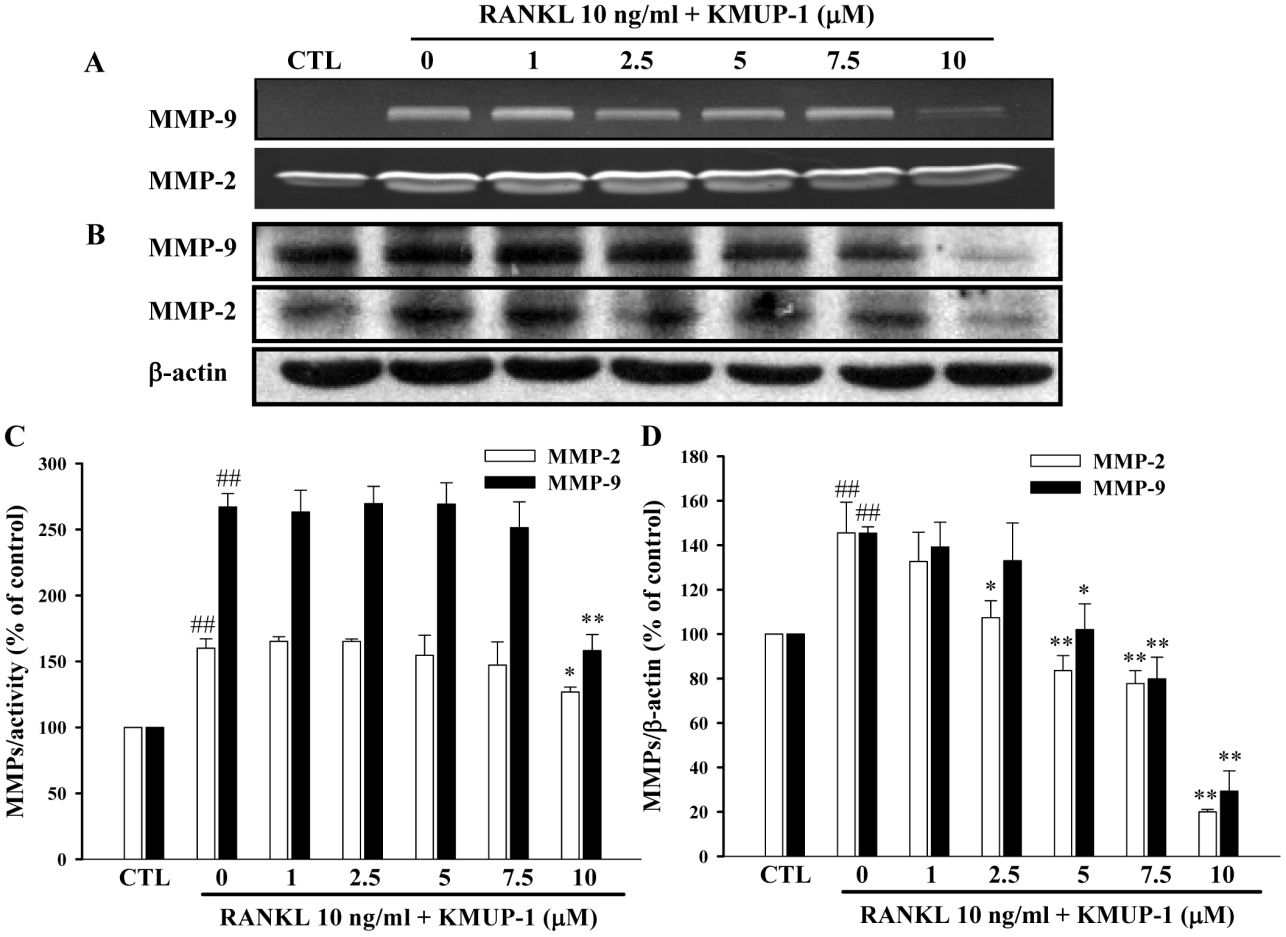
Effects of KMUP-1 on RANKL-induced activations of MMP-2 and MMP-9. RAW264.7 cells were cultured for 24 h in the presence of RANKL (10 ng/ml) and KMUP-1 (0–10 µM). (**A**, **C**) Effects on activities of MMP-2 and MMP-9 were analyzed by gelatin zymography. (**B**, **D**) Effects on protein expressions of MMP-2 and MMP-9 were analyzed by Western blotting. Each value represents the mean ± S.E.M. of three independent experiments, with triplicate determinations in each experiment. ^##^ *P*<0.01 compared with control; *P<0.05, **P<0.01 compared with RANKL alone.

Since MMP-2 and MMP-9 are thought to be important in mediating mobility of preosteoclasts and osteoclasts, we examined whether KMUP-1 can also decrease their activations induced by RANKL. We first found that RANKL induced increased MMP-9 mRNA levels approximately 4.63-fold than control, and KMUP-1 attenuated this effect concentration-dependently (Figure 5H). We next found parallel effects on protein expression of MMP-2 and MMP-9 (Figure 6B and 6D). In addition, RANKL-induced MMP-2 and MMP-9 activity was repressed by KMUP-1 at high concentration (Figure 6A and 6C).

### Effects of KMUP-1 on HMGB1 Translocation and Release

**Figure 7:**
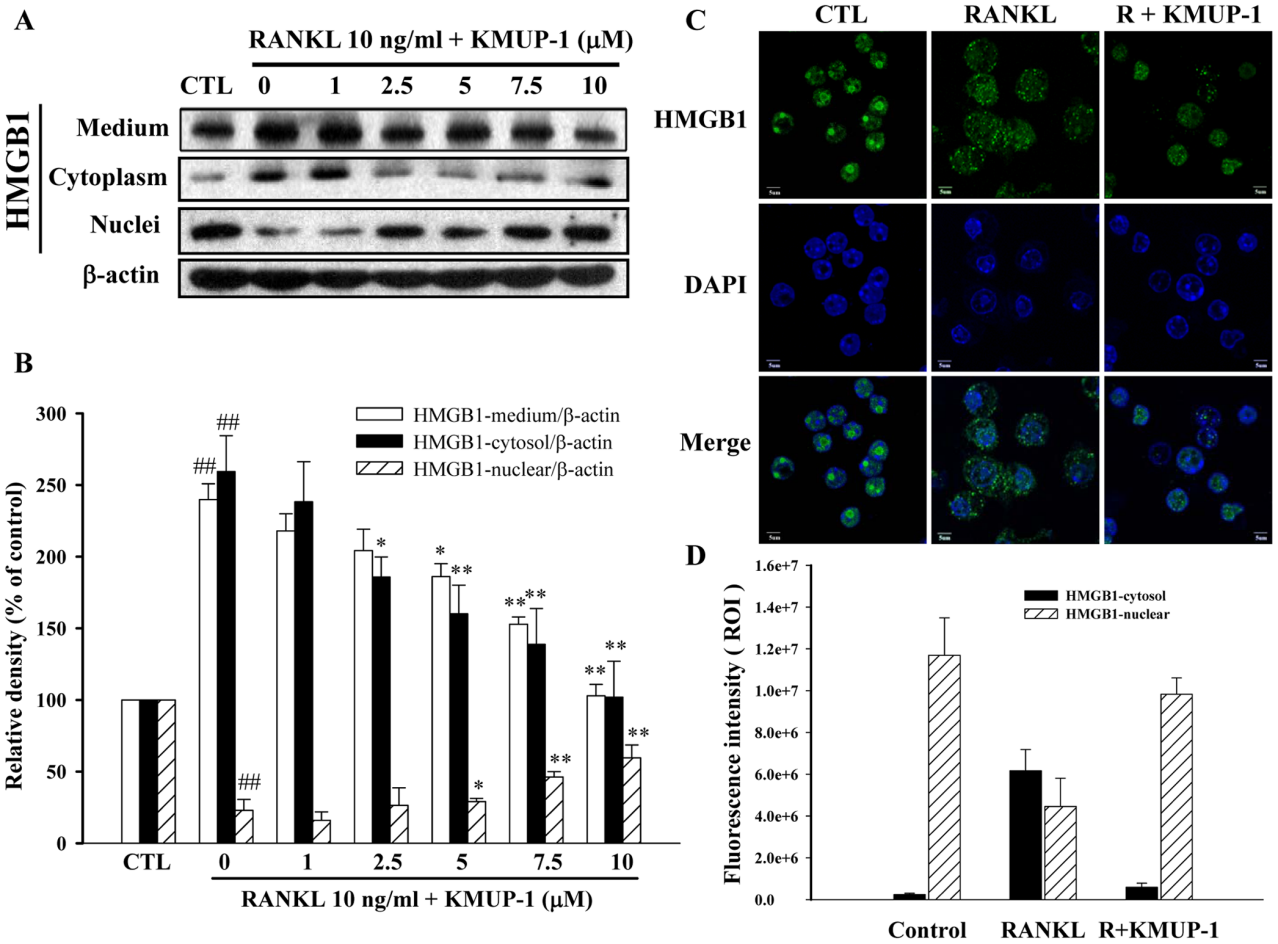
Effects of KMUP-1 on RANKL-induced HMGB1 translocation and release. (**A, B**) After culture for 18 h in the presence of RANKL (10 ng/ml) and KMUP-1 (0–10 µM), both the medium and cell lysates (nuclear/cytosol extracts) were immunoblotted against HMGB1 antibody. (**C, D**) Nuclear translocation of HMGB1 was also examined by confocal microscope. Scale bar: 5 µm. Each value represents the mean ± S.E.M. of three independent experiments, with triplicate determinations in each experiment. ^##^ *P*<0.01 compared with control; *P<0.05, **P<0.01 compared with RANKL alone.

It has been demonstrated that RANKL induces HMGB1 release during an early stage of differentiation from bone marrow-derived macrophages (BMMs) into osteoclasts, and extracellular HMGB1 acts as an osteoclastogenic cytokine by promoting cytokeleton reorganization and integrin signaling in RANKL-stimulated BMMs [25]. We therefore determined whether similar effects could be observed in RAW264.7 cells, and found that treatment with RANKL for 18 h increased levels of HMGB1 in media and translocation from the nuclear to the cytoplasmic compartment (Figure S1). KMUP-1 (5–10 µM) not only inhibited RANKL-induced HMGB1 cytosolic translocation as shown by Western blotting (Figure 7A and 7B) and immunocytochemistry (Figure 7C and 7D), and but also attenuated extracellular release of HMGB1 (Figure 7A and 7B).

### Effects of KMUP-1 on Signal Transduction Induced by RANKL

**Figure 8:**
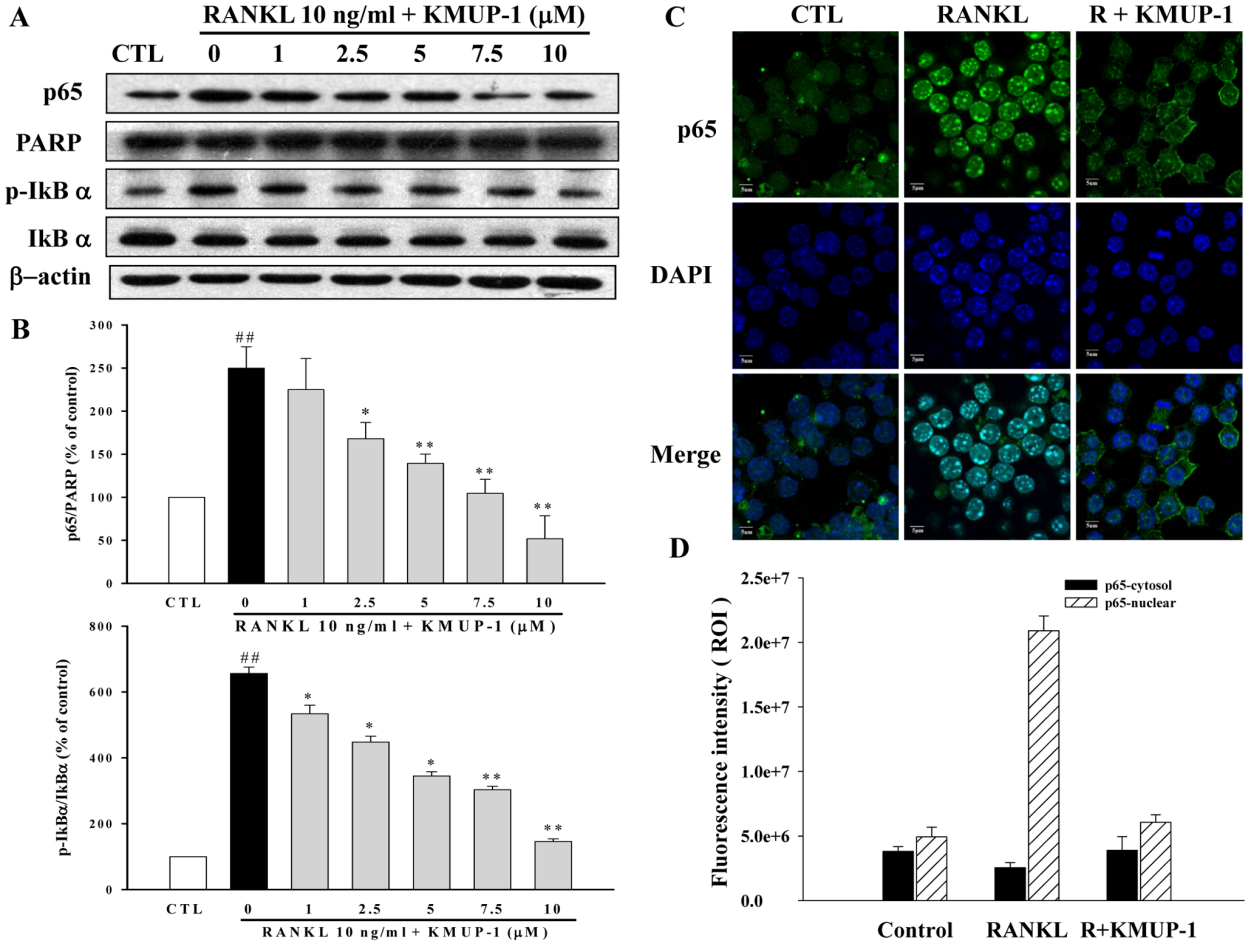
Effects of KMUP-1 on RANKL-induced NF-κB activation. Cells were pretreated with KMUP-1 (0–10 µM) for 24 h before the stimulation by RANKL (10 ng/ml) for 30 min. (**A**) Using Western blotting, the nuclear fractions were analyzed for protein content of p65, a subunit of NF-κB protein, and (**B**) the cytosolic fractions were analyzed for protein content of IκB-α and phosphorylated IκB-α. (**C, D**) Confocal microscopy demonstrated that KMUP-1 inhibited RANKL-induced nuclear translocation of p65 as shown by the location of anti-p65 stain within the nucleus stained with DAPI. Scale bar: 5 µm. Each value represents the mean ± S.E.M. of three independent experiments, with triplicate determinations in each experiment. ^##^ *P*<0.01 compared with control; *P<0.05, **P<0.01 compared with RANKL alone.

**Figure 9:**
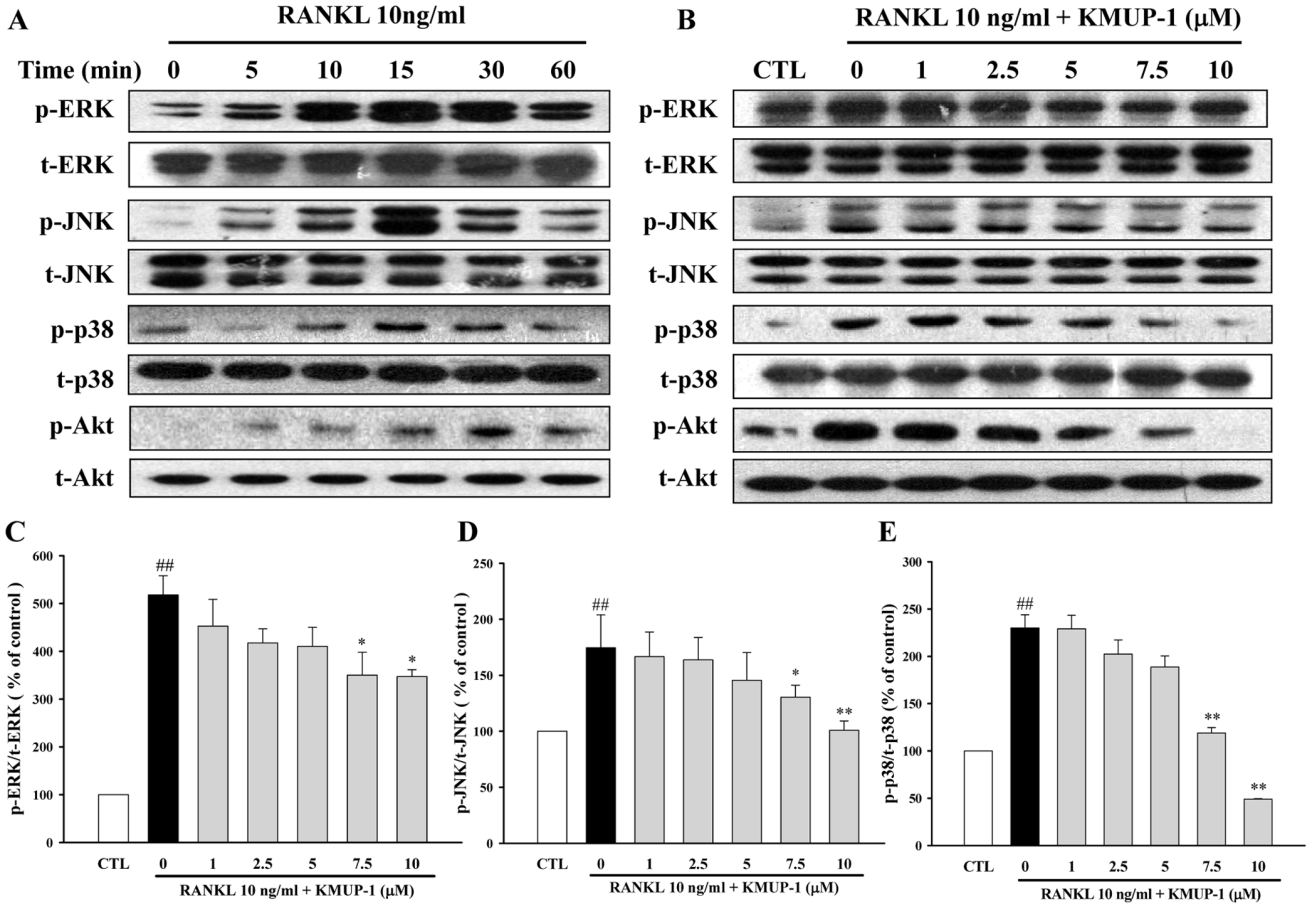
Effects of KMUP-1 on RANKL-induced activations of MAPKs and Akt pathways. (**A**) Time course analysis of RANKL-induced phosphorylation of MAPKs and Akt showed that their activations were maximized at 15 min or 30 min, respectively. (**B**–**E**) RAW264.7 cells were pretreated with KMUP-1 for 24 h followed by stimulation with RANKL (10 ng/ml) for 15 min (MAPKs) or 30 min (Akt). The cell lysates were analyzed by Western blotting. Each value represents the mean ± S.E.M. of three independent experiments, with triplicate determinations in each experiment. ^##^ *P*<0.01 compared with control; *P<0.05, **P<0.01 compared with RANKL alone.

To elucidate the molecular mechanisms underlying the inhibitory effects of KMUP-1 on osteoclast differentiation, we assessed the activations of NF-κB, MAPKs and Akt pathways. By both Western blotting and confocal microscope, we demonstrated that pre-treatment with KMUP-1 inhibited RANKL-induced nuclear translocation of p65, a subunit of NF-κB, in a concentration-dependent manner (Figure 8A–D). Similarly, pre-treatment with KMUP-1 inhibited RANKL-induced phosphorylation of IκB-α (Figure 8A and 8B). Furthermore, we found that RANKL activated MAPKs and Akt maximally at 15 min and 30 min, respectively (Figure 9A), and these activations were attenuated by KMUP-1 concentration-dependently (Figure 9B–E).

### Effects of KMUP-1 on c-Fos and the Calcium/Calcineurin/NFATc1 pathway

**Figure 10:**
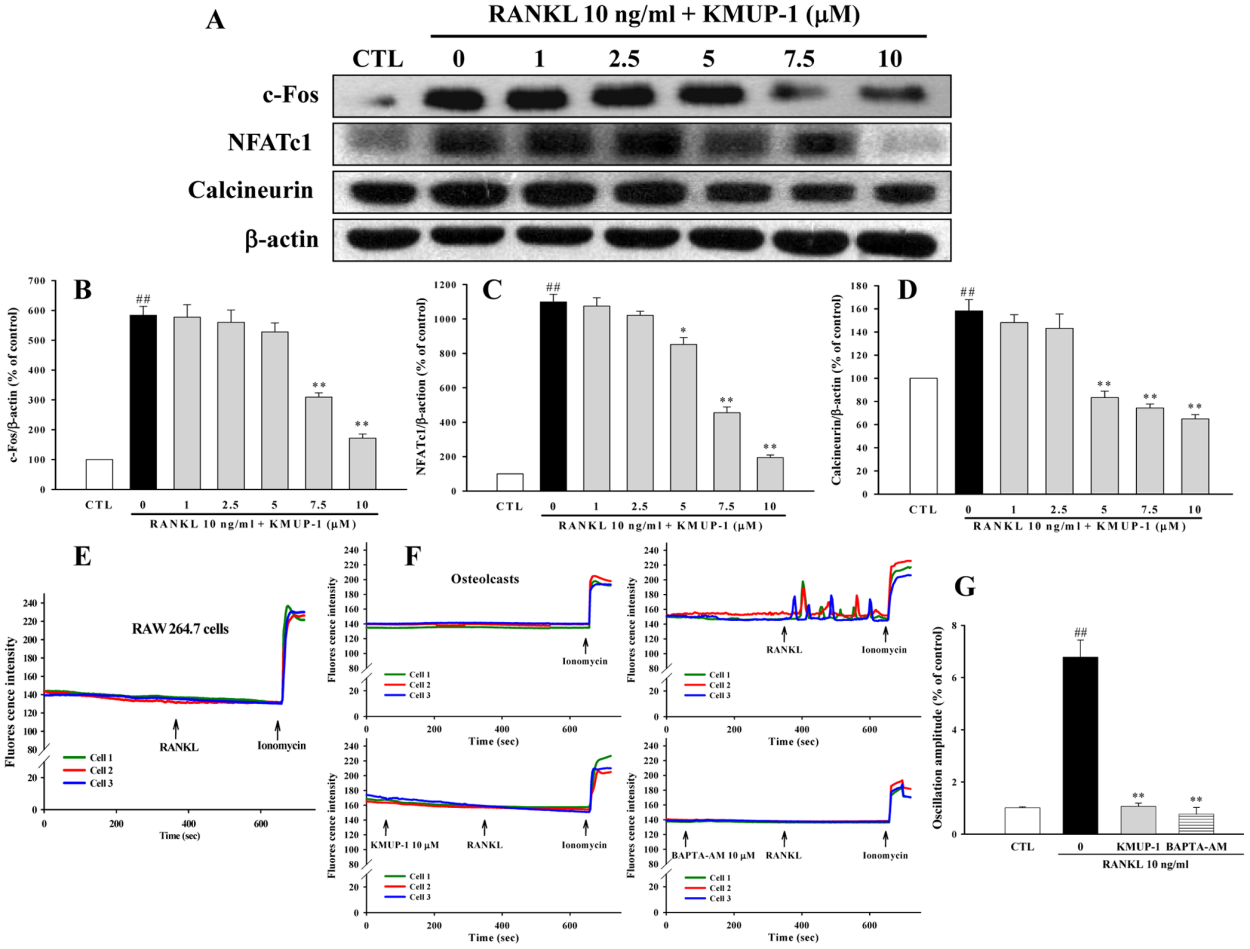
Effects of KMUP-1 on RANKL-induced activations of c-Fos and the calcium/calcineurin/NFATc1 pathway. (**A**) RAW264.7 cells were cultured for 24 h with RANKL (10 ng/ml) and KMUP-1 (0–10 µM). Cell lysates were then analyzed by Western blotting with antibodies against c-Fos, NFATc1, calcineurin and actin. (**B**–**D**) The expressions of these proteins were quantified by densitometry. (**E**) In RAW264.7 cells, RANKL did not induce Ca^2+^ oscillation. Each color indicates an individual cell in the same field. (**F, G)** In osteoclasts, KMUP-1 inhibited Ca^2+^ oscillation evoked by RANKL. Pretreatment with KMUP-1 (10 µM), similar to calcium chelator BAPTA (10 µM), significantly reduced the amplitude of oscillation induced by RANKL. Each value represents the mean ± S.E.M. of three independent experiments, with triplicate determinations in each experiment. ^##^ *P*<0.01 compared with control; *P<0.05, **P<0.01 compared with RANKL alone.

C-Fos and NFATc1 are both crucial transcription factors in osteoclastogenesis induced by RANKL [26]–[28]. Since KMUP-1 could inhibit mRNA of c-Fos and NFATc1 (Figure 5D and 5F), we further examined if there were parallel effects on their protein expression. As expected we found that RANKL up-regulated protein expressions of c-Fos and NFATc1, and these effects were attenuated by KMUP-1 (Figure 10A–C). Give that Ca^2+^/calcineurin pathway is critical for NFATc1-dependent osteoclastogenic gene transcription [29], we continued to perform experiments of Ca^2+^ signaling and calcineurin expression. We found that KMUP-1 down-regulated RANKL-induced expression of calcineurin concentration-dependently (Figure 10A and 10D). In addition, our results showed that in RAW264.7 cells, RANKL induced no significant Ca^2+^ oscillation (Figure 10E); however in osteoclasts, RANKL induced markedly Ca^2+^ oscillation (Figure 10F). Notably, KMUP-1, similar with calcium chelator BAPTA, prevented RANKL-induced Ca^2+^ oscillation (Figure 10F and G).

### Effects of KMUP-1 on Ovariectomy-induced Bone Loss

**Figure 11:**
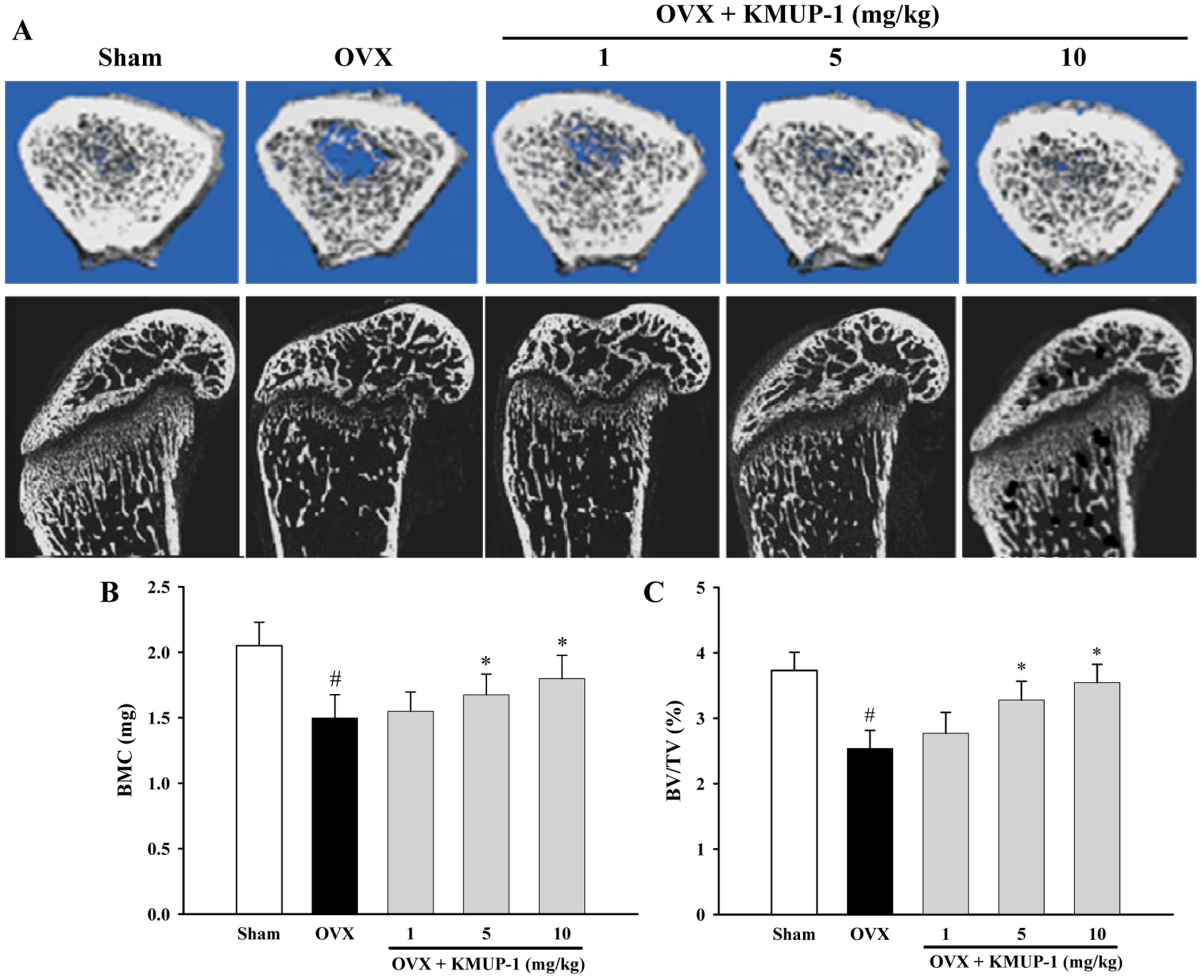
Effects of KMUP-1 on bone loss in ovariectomized (OVX) mice. (**A**) OVX mice were sacrificed after 30 days of KMUP-1 treatment. Images of the longitudinal and transverse sections of the proximal tibia were obtained with a µCT. (**B**, **C**) Tibial trabecular bone mineral content (BMC) and bone volume/tissue volume (BV/TV, %) were quantified from data obtained by the µCT. All values are expressed as mean ± S.E.M. ^#^ *P*<0.05 compared with the Sham group;^ *^ *P*<0.05 compared with the OVX group.

**Figure 12:**
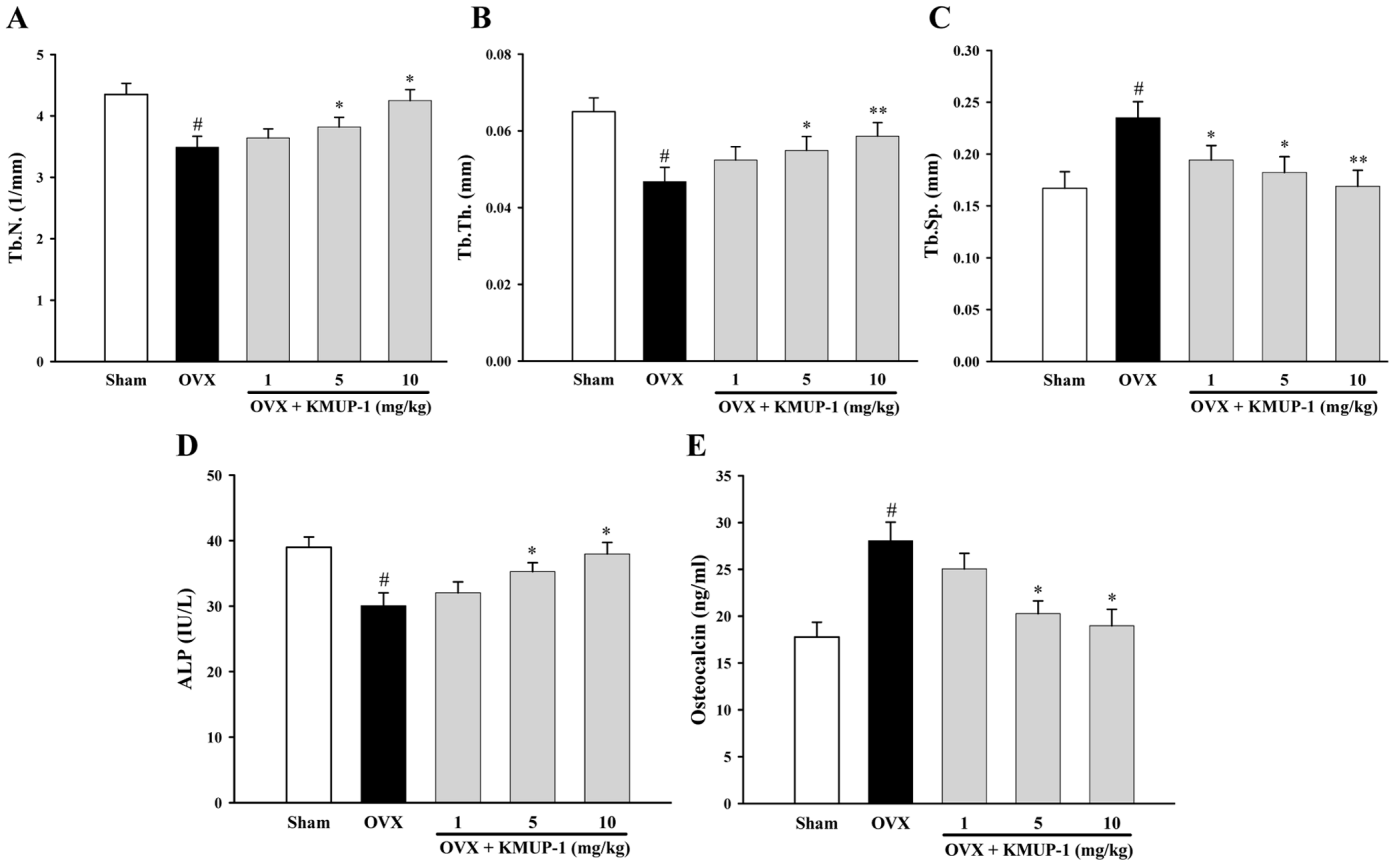
Effects of KMUP-1 on changes in histomorphometric and biochemical markers of bone turnover in OVX mice. Histomorphometric data and serum biochemical markers were compared in sham-operated, OVX mice, and OVX+KMUP-1 mice. (**A**) Tb.N, trabecular number, (**B**) Tb.Th, trabecular thickness, (**C**) Tb.Sp, trabecular separation, (**D**) ALP, serum alkaline phosphatase and (**E**) osteocalcin. All values are expressed as mean ± S.E.M. ^#^ *P*<0.05 compared with the Sham group;^ *^ *P*<0.05, ^**^ *P*<0.01 compared with the OVX group.

To examine the *in vivo* effect of KMUP-1 on ovariectomy-induced bone loss, we used µCT to analyze tibia of OVX mice. The µCT at metaphyses of the proximal tibia revealed that KMUP-1 inhibited bone loss induced by OVX (Figure 11A). In addition, KMUP-1 dose-dependently preserved the trabecular bone mineral content (BMC) and trabecular bone volume (BV/TV) in OVX mice (Figure 11B and 11 C). Similarly, KMUP-1 preserved trabecular number (Tb.N.) and trabecular thickness (Tb.Th.), and reduced trabecular separation (Tb.Sp.) in OVX mice (Figure 12A–C). Finally, OVX caused decrease of serum ALP and increase of osteocalcin, and KMUP-1 counteracted these effects dose-dependently (Figure 12D and 12 E).

## Discussion

Bone metabolism is regulated by a balance between formation of new bone by osteoblasts and resorption of old bone by osteoclasts. In this balance, RANKL provides an essential signal for osteoclastic differentiation during osteoclastogenesis. In this study, we report for the first time the anti-osteoclastogenic activity of a chemical synthetic xanthine-based derivative, KMUP-1. We found that KMUP-1 effectively suppressed RANKL-induced osteoclast differentiation *in vitro*. We further characterized its effects on expression of key regulators of osteoclast differentiation and associated target genes. In addition, its anti-osteoclastogenic activity is and mediated in part by cAMP and cGMP pathways with associated suppressing effects on the NF-κB, MAPKs, PI3K/Akt and calcium/calcineurin/NFATc1 pathways. Finally, we found that it also attenuated OVX-induced osteoclastogenesis and prevented bone loss *in vivo*.

Osteoclasts are multinucleated giant cells present only in bone, with the capacity to enhance bone resorption [29]. It is generally accepted that two critical steps contribute to osteoclastogenesis: 1) commitment of the progenitor cells to osteoclast precursor cells, which involves turn-on of osteoclast marker genes such as TRAP, and 2) fusion of the TRAP-positive mononuclear cells to form multinucleated osteoclasts. As shown in Figure 2, KMUP-1 inhibited TRAP activity and transformation of RAW264.7 cells into TRAP-positive multinucleated osteoclasts. Additionally, the pit formation assay revealed that KMUP-1 inhibited the bone resorptive activity of mature osteoclasts (Figure 3). We also observed that a high concentration of KMUP-1 (up to 10 µM) did not show any cytotoxicity in RAW264.7 cells (Figure 1A). Taken together, these results suggest that the inhibitory effect of KMUP-1 on osteoclastogenesis is due to its unique effect on osteoclast differentiation but not cytotoxicity.

Although the mechanisms by which KMUP-1 inhibits osteoclastogenesis are not fully understood, a potential mechanism is to inhibit production of pro-inflammatory mediators. While the RANK-RANKL signal is required for osteoclastogenesis, the efficiency of this process is influenced by cytokines. The proinflammatory cytokines including TNF-α, IL-1, and IL-6 augment osteoclastogenesis [30], while IL-10 antagonizes this process [31]. This study shows the inhibitory effects of KMUP-1 on osteoclastogenic proinflammatory cytokines, as evidenced by reduced production of RANKL-induced IL-1β, IL-6 and TNF-α in RAW264.7 cells (Figure 4). HMGB1, a nuclear protein released from activated macrophages or injured cells, has been implicated as an important pro-inflammatory mediator in sepsis, arthritis, and tumor-associated inflammation [32]. It has been demonstrated that release of extracellular HMGB1 can be induced by RANKL during an early stage of differentiation from BMMs into osteoclasts, and HMGB1 is believed as a critical mediator participating in RANKL-induced and integrin-dependent osteoclastogenesis [25]. The present study indicates that KMUP-1 not only inhibits RANKL-induced proinflammatory cytokines, but also inhibits extracellular release and cytoplasmic translocation of HMGB1 (Figure 7).

We next examined the effects of KMUP-1 on mRNA expression of osteoclastogenesis-related genes (TRAF6, TRAP, MMP-9, Fra-1, Fra-2, c-src and cathepsin K) and transcription factors (c-Fos and NFATc1). We found that KMUP-1 attenuated RANKL-induced mRNA expression of TRAP, MMP-9, Fra-1, cathepsin K, c-Fos and NFATc1 (Figure 5). In addition, these effects of KMUP-1 were mitigated by a PKA inhibitor and a PKG inhibitor, suggesting that they are mediated, at least in part, by cAMP and cGMP. These results are in line with our previous studies demonstrating that KMUP-1 can increase intracellular cAMP and cGMP in various types of cells [14]–[16], and strengthen the notion that PDEs may protect against bone loss via cAMP and cGMP pathways [6]–[10].

Among MMPs, MMP-9 is the most abundant gelatinolytic one in osteoclasts and has a major role in the invasive activity of osteoclasts [33]. It is also involved in regulating gene expression of osteoclast maturation makers, including TRAP and cathepsin K. Early mammalian osteoclast precursors express MMP-9 as well as TRAP, and as these precursors differentiate and become committed to the osteoclast lineage, they express high levels of several proteins such as c-Src, a marker for the osteoclast phenotype [34]. Our results further showed that KMUP-1 could suppress the RANKL-induced protein expression and enzyme activity of MMP-9 (Figure 6), indicating that KMUP-1 does affect the maturation stage of osteoclastogenesis.

The binding of RANKL to its receptor RANK, a member of the TNF receptor (TNFR) superfamily, recruits and induces the trimerization of adaptor molecules such as TRAF6. It subsequently leads to the activation of phosphatidylinositol 3-kinase (PI3K), serine/threonine kinase Akt, MAPKs and transcription factors such as NF-κB [35], [36]. In this study, KMUP-1 inhibited RANKL-induced activation of Akt and MAPKs (Figure 9) and also prevented the translocation of the NF-κB p65 subunit with the blockade of phosphorylation-induced degradation of the inhibitory partner, IκBα (Figure 8). Even though the exact mechanisms underlying how KMUP-1 affects these signaling molecules remain unclear, several lines of evidence suggest that cAMP and cGMP may have a role. For example, a recent study demonstrates that cAMP/PKA signaling can suppress RANKL-induced osteoclast differentiation through inhibition of NF-κB nuclear translocation and modulation of ERK [37]. Another study indicates that sildenafil, an inhibitor of PDE5A, can suppress activations of PI3K/Akt and calcineurin/NFAT signaling through the cGMP/PKG pathway [38]. Therefore, it is possible that in the present study cAMP and cGMP are both second messengers responsible these wide spectrum of effects exerted by KMUP-1.

The ovariectomized mouse has been widely used an animal model of postmenopausal osteoporosis with estrogen insufficiency. Finally, we used a mouse-OVX model which displays marked loss of the trabecular bone [39], and showed that KMUP-1 improves trabecular tibia BMC and parameters of bone loss (BV/TV, Tb.N., Tb.Sp. and Tb.Th.) (Figures 11 and 12). The loss of bone mass and the deterioration of bone microstructure have been linked to an imbalance between bone formation and bone resorption [40]. Biochemical markers of bone turnover such as serum ALP and osteocalcin have been widely used to measure the status of bone remodeling [41]. Our results showed that OVX caused increased bone turnover as evidenced by the decrease of ALP and the increase of osteocalcin. KMUP-1 dose-dependently reversed these effects, indicating that KMUP-1 prevents bone loss through inhibition of bone turnover.

In conclusion, the present study indicates that the PDE inhibitor KMUP-1 can inhibit osteoclastoogenesis of RAW264.7 cells and bone resorptive activity of mature osteoclasts. It can attenuate RANKL-induced mediators (cytokines and HMGB1) and signal transduction (Akt, MAPKs and calcium/calcineurin), regulate the expression of osteoclast-associated genes (TRAP, MMP-9, Fra-1, cathepsin K) and modulate the activation of transcriptional factors such as NF-κB, AP-1 and NFATc1. These effects are mediated, at least in part, by cAMP and cGMP pathways. Notably, it can prevent the decrease of bone mass and suppress bone turnover in OVX mice. Therefore, KMUP-1 may have a role in pharmacologic therapy of osteoporosis.

## Supporting Information

Figure S1 **The time course analysis of RANKL-induced HMGB1 expression.** RAW264.7 cells were stimulated with RANKL (10 ng/ml) for the indicated time. The expression of HMGB1 was examined by Western blot. Each value represents the mean ± S.E.M. of three independent experiments, with triplicate determinations in each experiment. ^##^ *P*<0.01 compared with control.
(TIF)
