# Supplementary figures and images for: KMUP-1 Suppresses RANKL-Induced Osteoclastogenesis and Prevents Ovariectomy-Induced Bone Loss: Roles of MAPKs, Akt, NF-κB and Calcium/Calcineurin/NFATc1 Pathways
Source: PLoS One. 2013 Jul 25;8(7):e69468. doi: 10.1371/journal.pone.0069468 (PMC3723916; doi:10.1371/journal.pone.0069468)

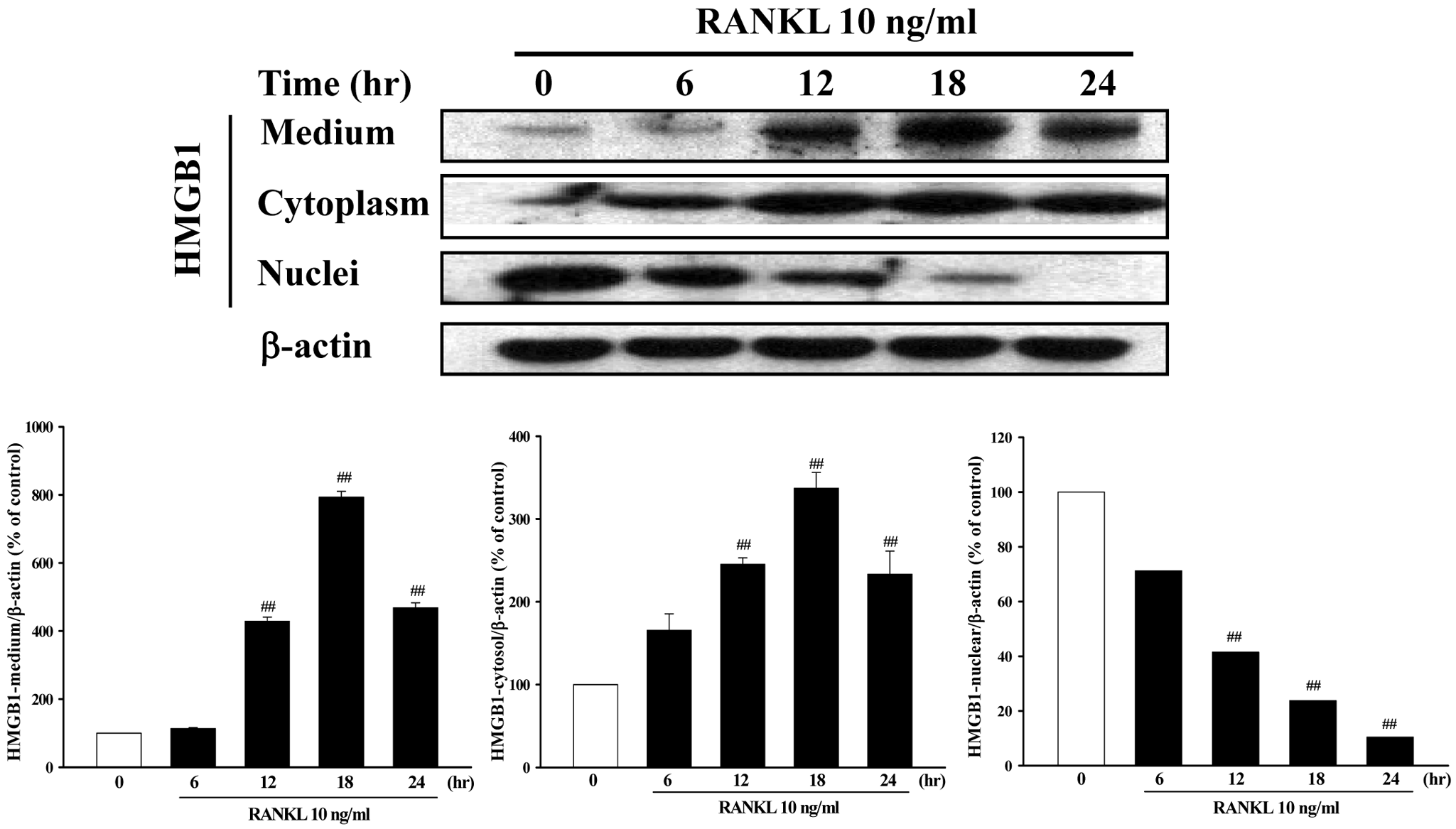

Supplement: Figure S1 — The time course analysis of RANKL-induced HMGB1 expression. RAW264.7 cells were stimulated with RANKL (10 ng/ml) for the indicated time. The expression of HMGB1 was examined by Western blot. Each value represents the mean ± S.E.M. of three independent experiments, with triplicate determinations in each experiment. ## P<0.01 compared with control. (TIF) [file pone.0069468.s001.tif]
